# An Asian study on clinical and psychological factors associated with personal recovery in people with psychosis

**DOI:** 10.1186/s12888-019-2238-9

**Published:** 2019-08-22

**Authors:** Madeline Lim, Ziqiang Li, Huiting Xie, Bhing Leet Tan, Jimmy Lee

**Affiliations:** 1 Research Division, Institute of Mental Health, Singapore, Singapore; 2 Department of Nursing, Institute of Mental Health, Singapore, Singapore; 3 Department of Occupational Therapy, Institute of Mental Health, Singapore, Singapore; 4 Singapore Institute of Technology, Singapore, Singapore; 5 Lee Kong Chian School of Medicine, Nanyang Technological University, Singapore, Singapore; 6 Department of Psychosis, Institute of Mental Health, 10 Buangkok View, Singapore, 539747 Singapore

**Keywords:** Recovery, Outcome, Hope, Psychosis, Schizophrenia, CHIME, QPR

## Abstract

**Background:**

Despite the rising recognition of personal recovery, there is a lack of research on personal recovery in individuals with psychosis in Singapore. This study aims to evaluate the psychometric properties of the QPR-15 using the CHIME personal recovery framework and to examine its associations with clinical recovery factors.

**Methods:**

Sixty-six stable outpatients were recruited and assessed at two time points approximately 2 weeks apart. Convergent validity was examined through Spearman correlations with scores on CHIME-related psychological factors: connectedness (Ryff subscale- positive relations with others), hope (Herth Hope Index- abbreviated), identity (Ryff subscale- self-acceptance, Internalized Stigma of Mental Illness- Brief), meaning (World Health Organization Quality of Life Assessment-Brief Form), empowerment (Empowerment Scale). Pearson’s correlation was used to examine the test-retest reliability, while Cronbach’s alpha was used to examine internal consistency. The initial factor structure was evaluated via principal component analysis, Velicer’s minimum average partial (MAP) criteria, parallel analysis, and a scree plot. Spearman correlations and hierarchical multiple linear regression (controlling for age and gender) were employed to examine the association of clinical (symptoms and functioning) and psychological factors with the QPR-15.

**Results:**

The QPR-15 demonstrated convergent validity with all CHIME-related psychological factors (r_s_ ranged from 0.472 to 0.687). Internal consistency was excellent (Cronbach’s alpha = 0.934), and test-retest reliability was adequate (r = 0.708). Initial factor structure evaluations revealed a one-factor model. Correlations of clinical factors with the QPR-15 were mostly low (r_s_ ranged from − 0.105 to − 0.544) but significant, except for depressive symptoms (CDSS: r_s_ = − 0.529 to − 0.544), while correlations were moderate for psychological factors. Clinical factors significantly explained 28.3–31.8% of the variance of the QPR-15. Adding psychological factors significantly increased the model variance at baseline (∆ adjusted R^2^ = 0.369, F change < 0.001) and at time point 2 (∆ adjusted R^2^ = 0.208, F change < 0.001).

**Conclusion:**

Our results provide preliminary evidence that the QPR-15 has adequate psychometric properties in Singapore and encompasses the CHIME personal recovery framework. In addition, our results suggest that clinical recovery and personal recovery are not substitutes for each other but rather are complementary, thereby promoting a more holistic evaluation of recovery in people with psychosis. Implications are discussed.

**Electronic supplementary material:**

The online version of this article (10.1186/s12888-019-2238-9) contains supplementary material, which is available to authorized users.

## Background

The concept of recovery in mental health has been re-examined, and recent developments have revealed two non-synonymous recovery constructs: clinical recovery (objective state, i.e., symptoms, functioning) and personal recovery (subjective process, i.e., attitude or life orientation) [1–4]. Findings from a previous study with 381 service users substantiate the importance of personal recovery, as it was found that the highest level of consensus on the definition of recovery was “recovery is the achievement of a personally acceptable quality of life” and “recovery is feeling better about yourself” [5]. Therefore, this angle of recovery may only be assessable by the individuals themselves and may approximate us to a more holistic definition of recovery. Moreover, personal recovery impacts the quality of life of individuals with schizophrenia [6], and a reduction in psychiatric symptoms alone does not necessarily translate into higher personal recovery [7, 8]. Hence, it may be essential to consider personal recovery in the evaluation of overall recovery in people with psychosis.

Although it may be understood in clinical practice that some form of clinical recovery is essential for personal recovery to take place, the recovery movement has contested that personal recovery can take place without clinical recovery [9]. In a recent study, Macpherson et al. [10] administered a series of measures and found that these measures eventually clustered into three factors (termed patient-rated personal recovery, patient-rated clinical recovery, and staff-rated clinical recovery). Although only patient-rated personal recovery (consisting of measures of personal recovery (QPR), empowerment, well-being and hope) had improved over the course of 1 year, closer examination shows that the change in patient-rated personal recovery scores was significantly correlated with the change in symptom scores. This finding suggested that clinical factors should not be forsaken, as they can affect personal recovery. There were other similar findings in the literature, as certain clinical factors were found to be correlated with [3] and predictive of [11] personal recovery as well as of recovery orientation [7]. Clarifying the relationship between these two forms of recovery is essential for appropriate subsequent interventions, as we could promote synergy between the two [12] instead of having a trade-off based on their relative importance.

While it remains challenging to quantify personal recovery, several reviews have uncovered broad themes that are common in these subjective accounts of recovery [13–16]. Accordingly, several scales that aim to measure personal recovery have been developed, and these scales have been comprehensively reviewed within the previous two decades [17–22]. In 2011, Leamy and colleagues [23] adopted a systematic review and narrative synthesis approach to identify a conceptual framework that describes the process of recovery: connectedness; hope and optimism about the future; identity; meaning in life; and empowerment (CHIME). In contrast, with other frameworks that have been developed from limited samples or types of data, this framework was derived from 97 studies that consist of wider data sources, providing an empirical basis for related future work. Since then, the Questionnaire about the Process of Recovery (QPR) has been identified as the personal recovery measure that most closely maps to the CHIME framework [24].

To date, six studies have evaluated the psychometric properties of the QPR and have shown that it has good internal consistency, construct validity and test-retest reliability [25–30]. However, Williams and colleagues [27] found that the interpersonal subscale underperforms in terms of its psychometric properties and that the intrapersonal subscale overlaps substantially with the 15-item version (see methods for information on the QPR). Hence, they have recommended the 15-item version, as it is more robust and less burdensome. Two other studies had similar findings. Law and colleagues [28] found that the original 2-factor solution fit their data poorly and that the internal consistency could be improved by the removal of seven items. Similarly, Argentzell et al. [25] found that the psychometric properties of a brief Swedish QPR-16 version were better supported, where the items of factor 2 (interpersonal) had underperformed. With these findings, the shorter QPR-15 version may be a better choice in terms of psychometric properties and feasibility for clinical use.

The six existing QPR studies, however, have not been able to confirm the conclusion of Shanks and colleagues that the QPR maps to the CHIME framework most closely, as the CHIME framework has not been completely represented in these studies. Given that the current literature has not been comprehensive in its empirical method of testing, it remains uncertain if the QPR encompasses the CHIME framework, let alone the QPR-15 (shortened version).

Moreover, no studies on personal recovery from mental illnesses have been conducted in Singapore, as it is a relatively new concept in Asia [31]. However, it was reported that Western concepts of personal recovery were applicable in Hong Kong [32–34], an Asian socio-cultural setting. Singapore is a country with a multi-ethnic and predominantly Asian society, with English as the main language of communication. Before the move towards recovery-oriented mental health services can occur, the applicability of valid personal recovery measures in our socio-cultural setting is one of the steps required. Furthermore, more recovery research has been encouraged in culturally dissimilar societies [35].

Therefore, one of the aims of the current study is to evaluate the psychometric properties of the QPR-15 in Singapore. We aim to test its validity using the CHIME framework and to examine its initial factor structure and its reliability in terms of internal consistency and test-retest reliability. In addition, we aim to examine the associations of personal recovery with clinical and psychological factors, as it remains unclear how the two forms of recovery should be viewed and applied clinically. Although two QPR studies [8, 28] have included clinical factors, the authors did not enter them into a single model to represent clinical recovery in the analysis. We hypothesise that compared to psychological factors, clinical factors will have significant but lower associations with personal recovery.

## Method

### Participants

Participants were outpatients recruited from the Institute of Mental Health (IMH) in Singapore. Inclusion criteria were (a) diagnoses of schizophrenia and schizoaffective disorders, (b) ability to speak and understand English well enough to complete the measures, (c) ability to give informed consent, and (d) age between 21 and 65 years old. The exclusion criteria were (a) current illicit substance use, (b) neurological disorders, and (c) intellectual disability.

### Design and procedures

Two assessments were conducted, with the second assessment scheduled approximately 2 weeks after the baseline assessment. Diagnoses were ascertained using the Structured Clinical Interview for DSM-IV-TR Axis I Disorders in the first assessment. The baseline assessment included the following: socio-demographic information; the QPR-15; and clinical factors consisting of the Positive and Negative Symptoms Scale (PANSS), the Calgary Depression Scale for Schizophrenia (CDSS), and the Personal and Social Performance Scale (PSP). Due to concerns related to assessment burden, the assessment of psychological factors was separated between the two time points, approximately 2 weeks apart. Hence, the baseline psychological factors assessed were the Herth Hope Index-abbreviated (HHI- abbreviated), the Internalized Stigma of Mental Illness Scale-Brief (ISMI-Brief), and the Empowerment Scale. Psychological factors assessed at time point 2 were the World Health Organization Quality of Life Assessment-Brief Form (WHOQOL-BREF) and the subscales of self-acceptance and positive relations with others from the Ryff Scales of Well-Being (Ryff). The QPR-15 and the clinical scales that were assessed at the baseline assessment were also assessed at time point 2. The psychological factors selected are the constituents of the recovery process themed by Leamy et al. [23] as CHIME (refer to Table 2 of their paper). All assessors were trained in the conduct of the scales. Ethics approval was provided by the National Healthcare Group’s Domain-Specific Review Board. Informed consent from all participants was obtained prior to the baseline assessment.

### Measures

#### The questionnaire about the process of recovery (QPR-15)

The QPR is one of the tools developed in collaboration with service users through interviews to generate themes and items of the scale in the UK [29]. The items were generated based on the findings of Pitt et al.: themes of rebuilding life (subtheme: active participation in life, rebuilding social support), rebuilding self (subtheme: understanding of self, empowerment) and hope for a better future (subtheme: process of change, desire for change) [36]. Two service-user researchers were part of the research team, and a steering committee of 10 service users was consulted at each stage of the research [21]. Service users’ review of the measure was positive (easy and quick to complete, positive language and items, and items reflecting the wider aims of recovery) [21]. The initial 22-item version had two subscales: QPR intrapersonal and QPR interpersonal. The intrapersonal subscale refers to tasks that individuals are responsible for carrying out and that they complete to rebuild their lives, while the interpersonal subscale refers to individuals’ ability to reflect on their value in the external world and on how recovery is facilitated by external processes and interpersonal relationships with others [29]. The QPR-22, however, has since been revised to 15 items, with a one-factor solution called the QPR-total [28]. The evaluation of the 15-item version has shown adequate convergent validity (r = 0.73), test-retest reliability (r = 0.70–0.74), sensitivity to change (r = 0.40) and internal consistency (α = 0.93) [27, 28]. The QPR is a self-rated scale, scored using a 5-point Likert scale ranging from 0 (disagree strongly) to 4 (agree strongly).

#### Positive and negative syndrome scale (PANSS)

Symptoms were assessed on the PANSS by trained raters. The scale has been validated locally and found to comprise five factors [37]: positive, negative, excitement, depression and cognitive.

#### Calgary depression scale for schizophrenia (CDSS)

The CDSS is a rating scale developed for assessing the severity of depressive symptoms in schizophrenia [38]. It is a 9-item observer-rated measure with a global score range of 0–27. Its psychometric properties have been extensively explored [39–42], and it proves to be a reliable and valid tool.

#### Personal and social performance scale (PSP)

The PSP [43] is an observer-rated functioning scale, assessed through four domains: 1) socially useful activities, 2) personal and social relationships, 3) self-care and 4) disturbing and aggressive behaviours. The total score ranges from 0 to 100, with a higher score indicating higher functioning. Its validity, internal consistency, test-retest reliability and inter-rater reliability have been established [44–48]. It has been shown to have the ability to discriminate between groups of patients with different symptom severities [44, 45].

#### Herth Hope index (HHI)-abbreviated

The HHI-abbreviated version [49] is a 12-item self-rated scale that measures hope based on a) temporality and future, b) positive readiness and expectancy, and c) interconnectedness. It is scored using a four-point Likert scale ranging from 1 (strongly disagree) to 4 (strongly agree), in which a higher score is indicative of higher levels of hope. It has been demonstrated to have good reliability and test-retest reliability, as well as having criterion and divergent validity [49]. The scale’s internal consistency was also good (Cronbach’s α = 0.90) when used in our sample.

The HHI-abbreviated was selected to represent the domain of hope and optimism about the future of the CHIME recovery framework; 79% of the studies have reported hope and optimism about the future as part of the recovery process [23].

#### Internalized stigma of mental illness scale (ISMI)-brief

The 10-item ISMI [50] is a self-rated scale that measures internalized stigma with the following subscales: alienation, discrimination experience, social withdrawal, stereotype endorsement, and stigma resistance. It possesses adequate internal consistency and construct validity. In our sample, its internal consistency was good (Cronbach’s α = 0.81). It is scored on a 4-point Likert scale (1 = strongly disagree to 4 = strongly agree), with higher scores indicative of greater internalized stigma.

The ISMI-Brief was selected to represent the domain of identity of the CHIME recovery framework; 46% of studies have reported overcoming stigma as part of the recovery process [23].

#### Empowerment scale

The Empowerment Scale [51] is a 28-item self-rated instrument that measures empowerment as defined by consumers of mental health services, based on five factors: self-efficacy/self-esteem, power/powerlessness, community/activism, righteous/anger, and optimism/control over the future. It has high internal consistency and construct validity [51] and has been validated in an outpatient mental health population [52]. In our sample, its internal consistency was good (Cronbach’s α = 0.71). The scale is rated on a four-point Likert scale (1 = strongly disagree to 4 = strongly agree), with higher scores representing higher endorsement of empowerment.

The Empowerment Scale was selected to represent the domain of empowerment in the CHIME recovery framework; 91% of the studies reported empowerment as part of the recovery process [23].

#### World Health Organization quality of life assessment-brief form (WHOQOL-BREF)

The WHOQOL-BREF [53] is a 26-item self-rating scale that measures one’s subjective QOL based on four domains: physical health (7 items), psychological health (6 items), social relationships (3 items) and environment (8 items). It also contains two items assessing the overall perception of QOL and overall health satisfaction. The scale was developed based on the concept of QOL as “an individual’s perception of their position in life in the context of the culture and value systems in which they live and in relation to their goals, expectations, standards and concerns” (p.5) [53]. It has adequate internal consistency, discriminant validity (illness versus healthy samples), and validity, as a factor analysis supported a 4-factor structure [54]. In our sample, it has good internal consistency (Cronbach’s α = 0.94). It is rated on a five-point scale (1 = very poor, 2 = poor, 3 = neither poor nor good, 4 = good, and 5 = very good), with higher scores reflecting greater QOL.

The WHOQOL-BREF was selected to represent the domain of meaning in life in the CHIME recovery framework; 65% of the studies reported having quality of life as part of meaning in life [23]. Given that there is an item in the WHOQOL-BREF (item 6) that directly assesses meaning in life (“to what extent do you feel your life to be meaningful”), we will also use this single item to represent meaning in life itself, as 66% of the studies have reported this quality to be important in the recovery process.

#### Ryff scales of well-being

The Ryff Scales of Well-Being [55] measure psychological well-being (eudaimonia happiness). Two subscale scores were used: self-acceptance (9 items) and positive relations with others (9 items). The internal consistencies of these two subscales in our sample were good (self-acceptance: Cronbach’s α = 0.76; positive relations with others: Cronbach’s α = 0.80). It is rated on a scale of 1 (strongly disagree) to 6 (strongly agree). The self-acceptance subscale was selected to reflect the domain of identity in the CHIME recovery framework; 75% of the studies reported identity as important in the recovery process. Positive relations with others subscale was selected to reflect the domain of connectedness; 86% of the studies reported connectedness to be important in the recovery process [23].

### Statistical analyses

All data were analysed using IBM SPSS Statistics, version 23 (IBM SPSS, Armonk, N.Y., USA), except the post hoc power analysis, which was conducted on G*power (version 3.1) [56]. Total scores were used for all scales except the subscale scores of the Ryff Scales of Psychological Well-Being (self-acceptance and positive relations with others) and the single item score of the WHOQOL-BREF (item 6: meaning in life). Convergent validity with psychological factors was assessed using Spearman correlations, as the variables were of non-normal distribution. Reliability in terms of internal consistency and test-retest reliability were assessed. Internal consistency of items was assessed using Cronbach’s alpha coefficient. Corrected item-total correlations and Cronbach’s alphas if an item were deleted were also examined. Items were considered redundant if the item-total correlation was < 0.3 [57] or if deleted items would substantially increase the Cronbach’s alpha (> 0.5) [28]. Test-retest reliability was assessed using Pearson’s correlation on QPR-15 data collected at baseline and follow-up, as the data were normally distributed.

The initial factor structure of the QPR-15 was evaluated via principal component analysis. A scree plot, Velicer’s minimum average partial (MAP) criteria and parallel analysis were used to estimate the component structure.

Spearman correlations were used to evaluate the associations of the QPR-15 with psychological and clinical factors. The correlation coefficient was interpreted according to the following limits: ± 0–0.3 as little, ± 0.3–0.5 as low, ± 0.5–0.7 as moderate, ± 0.7–0.9 as high and ± 0.9–1 as very high [58]. Hierarchical multiple linear regression was used to examine the relationships among clinical factors, CHIME-related psychological factors and personal recovery. Psychological and clinical factors that had significant correlations with the QPR-15 were entered into the model, which was run separately for the two time points. This was a two-stage model with clinical factors entered in the first stage and psychological factors entered in the second stage. This sequence was determined based on the expectation that psychological factors would have higher predictive value for personal recovery [28]. To detect the issue of multicollinearity in hierarchical multiple regression, a Variance Inflation Factor (VIF) of 2 was chosen as a cut-off for stricter control of collinearity. Statistical significance was set at *p* < 0.05.

## Results

### Sample characteristics

The socio-demographic and clinical characteristics of the 66 participants are presented in Table 1. The descriptive statistics of the scores for the measures are summarised in Table 2.

**Table 1:** Sample characteristics (N = 66)

Characteristics	Mean (SD) or n (%)
	n (%)
Gender	
Male	30 (45.5)
Female	36 (54.5)
Ethnicity	
Chinese	61 (92.4)
Malay	2 (3.0)
Indian	2 (3.0)
Other	1 (1.5)
Marital Status	
Single	51 (77.3)
Married/living with someone as if married	9 (13.6)
Divorced	6 (9.1)
Living arrangement	
Living in a community home	12 (18.2)
Living alone	1 (1.5)
Living with family	48 (72.7)
Living with friends	5 (7.6)
Highest education level	
Junior college	8 (12.1)
Vocational training	20 (30.3)
Tertiary	38 (57.6)
Psychiatric diagnosis	
Schizophrenia	64 (97.0)
Schizoaffective disorder	2 (3.0)
Current employment	
Unemployed	26 (39.4)
Employed	40 (60.6)
	Mean (SD)
Age in years	40.29 (10.552)
Duration of illness, years	15.025 (9.215)
Antipsychotic dose, mg	449.243 (391.185)

Antipsychotic dose is in total daily CPZ mg equivalent

**Table 2:** Sample scores on study measures

	Mean	Std. deviation	Median	Range
Baseline assessment				
QPR-15	41.64	9.35	42.00	13.00–60.00
HHI-abbreviated	35.17	4.97	35.00	21.00–48.00
ISMI-Brief	2.34	0.50	2.30	1.20–3.50
Empowerment	69.55	5.95	70.00	56.00–88.00
PANSS positive	10.61	4.63	10.00	4.00–20.00
PANSS negative	8.00	3.36	7.00	5.00–20.00
PANSS excitement	4.18	1.76	3.00	3.00–11.00
PANSS depression	6.03	3.10	5.00	3.00–17.00
PANSS cognitive	4.42	1.30	5.00	2.00–7.00
CDSS	2.79	3.42	1.00	0.00–15.00
PSP	59.53	11.74	61.00	31.00–92.00
Time point 2 assessment				
QPR-15	43.30	8.76	44.00	14.00–60.00
WHOQOL-BREF	90.59	15.02	93.50	41.00–120.00
Physical health	13.87	2.64	14.29	5.71–17.71
Psychological health	13.70	2.60	14.67	6.67–18.00
Social relationships	13.62	3.00	14.67	4.00–20.00
Environment	14.31	2.25	14.50	7.50–20.00
Overall QOL	3.67	0.92	4.00	1.00–5.00
Overall health satisfaction	3.27	1.06	3.00	1.00–5.00
Item 6 of WHOQOL-BREF	3.33	0.98	3.00	1.00–5.00
Ryff self-acceptance	34.98	7.21	35.00	12.00–52.00
Ryff positive relations	35.55	7.68	37.00	20.00–51.00
PANSS positive	9.77	4.72	9.00	4.00–23.00
PANSS negative	8.32	3.40	7.50	5.00–20.00
PANSS excitement	4.58	2.20	4.00	3.00–15.00
PANSS depression	6.41	3.64	5.00	3.00–16.00
PANSS cognitive	4.44	1.51	5.00	2.00–9.00
CDSS	2.97	3.62	1.50	0.00–14.00
PSP	57.70	13.20	61.00	23.00–93.00

### Convergent validity

Spearman correlations between the QPR-15 and all other measures are shown in Table 3. The QPR-15 had significant and moderate associations with all of the CHIME themes that represent connectedness (Ryff positive relations with others: r_s_ = 0.633, *p* < 0.001), hope and optimism about the future (HHI: r_s_ = 0.687, *p* < 0.001), identity (ISMI: r_s_ = − 0.686, p < 0.001; Ryff self-acceptance: r_s_ = 0.521, *p* < 0.001), meaning in life (WHOQOL-BREF: r_s_ = 0.669, *p* < 0.001), and empowerment (Empowerment Scale: r_s_ = 0.547, p < 0.001). The only low correlation was with WHOQOL-BREF item 6 (CHIME theme of meaning in life; r_s_ = 0.472, p < 0.001).

**Table 3:** Associations of the QPR-15 with psychological and clinical factors

	Baseline QPR-15	Time point 2 QPR-15
HHI-abbreviated	0.687***	
ISMI-Brief	−0.686***	
Empowerment	0.547***	
PANSS positive	−0.311*	−0.348**
PANSS negative	−0.398**	−0.352**
PANSS excitement	−0.259*	−0.161
PANSS depression	−0.468***	−0.434***
PANSS cognitive	−0.105	−0.192
CDSS	−0.529***	−0.544***
PSP	0.355**	0.361**
WHOQOL-BREF		0.669***
WHOQOL-BREF item 6-meaningful		0.472***
Ryff self-acceptance		0.521***
Ryff positive relations		0.633***

Reported correlations are significant at the *p < 0.05, **p < 0.01 or *** *p* < 0.001 levels

### Reliability

Cronbach’s alpha coefficient for the QPR-15 items revealed excellent internal consistency (α = 0.934). The corrected item-total correlations and Cronbach’s alpha are shown in Table 4. There were no items that had negative correlations or corrected item-total correlations of below 0.3. In addition, none of the items, if removed, would lead to a substantial improvement in the Cronbach’s alpha of the measure as a whole (Items 4 and 15: increase in α by 0.001). Hence, no items were considered redundant. The QPR-15 was also found to have good test-retest reliability (r = 0.708, p < 0.001).

**Table 4:** Internal consistency and item loadings after rotation from factor analysis of the QPR-15

Item	Corrected-	Cronbach’s alpha	Two components	
	item correlation	if item deleted	Component 1	Component 2
1. I feel better about myself	0.793	0.926	0.829	0.149
2. I feel able to take chances in life	0.573^a^	0.932	0.633	0.125
3. I am able to develop positive relationships with other people	0.674	0.929	0.715	0.313
4. I feel part of society rather than isolated	0.478^a^	0.935	0.525	0.653
5. I am able to assert myself	0.601	0.932	0.640	0.498
6. I feel that my life has a purpose	0.810	0.925	0.846	0.003
7. My experiences have changed me for the better	0.669	0.929	0.722	−0.182
8. I have been able to come to terms with things that have happened to me in the past and move on with my life	0.678	0.929	0.722	0.141
9. I am basically strongly motivated to get better	0.783	0.926	0.831	−0.357
10. I can recognise the positive things I have done	0.699	0.928	0.758	−0.374
11. I am able to understand myself better	0.697	0.929	0.759	−0.453
12. I can take charge of my life	0.773	0.926	0.819	−0.058
13. I can actively engage with life	0.662	0.929	0.708	−0.032
14. I can take control of aspects of my life	0.808	0.926	0.845	0.054
15. I can find the time to do the things I enjoy	0.429^a^	0.935	0.482	−0.230

^a^Denotes corrected item correlation that fell below 0.6

### Initial factor structure

The item loadings are reflected in Table 5. As variance accounted for might be inflated with a small sample [59], this was not reported. On examination of the scree plot (Additional file 1: Figure S1), one component was suggested to be retained, but results from both Velicer’s MAP criteria and parallel analysis indicated the retention of two components. Because fewer than 3 items [59] had higher factor loadings on component 2, component 2 was not retained. Furthermore, all item loadings were > 0.4 for the first factor.

**Table 5:** Multiple regression analysis: predictors of recovery (QPR-15) scores

	Standardised β	*t*	*p*-value	F Change	Sig. F Change	
Baseline						
1						
Gender	0.025	0.231	0.818	5.335	< 0.001	
Age	0.102	0.969	0.337			
PANSS positive	−0.103	− 0.811	0.421			
PANSS negative	−0.293	−2.643	0.011			
PANSS excitement	−0.159	−1.499	0.139			
CDSS	−0.404	−3.286	0.002			
PSP	−0.074	− 0.556	0.580			
2						
Gender	0.058	0.794	0.430	69.332	< 0.001	
Age	0.080	1.119	0.268			
PANSS positive	−0.097	−1.132	0.262			
PANSS negative	−0.103	−1.311	0.195			
PANSS excitement	−0.085	−1.174	0.245			
CDSS	−0.219	−2.537	0.014			
PSP	−0.143	−1.539	0.130			
HHI-abbreviated	0.653	8.327	< 0.001			
Time point 2						
1						
Gender	−0.029	−0.271	0.787	4.661	< 0.001	
Age	0.191	1.745	0.086			
PANSS positive	−0.069	− 0.528	0.600			
PANSS negative	−0.125	−1.027	0.308			
PANSS excitement	0.023	0.198	0.844			
CDSS	−0.417	−3.384	0.001			
PSP	0.087	0.624	0.535			
2						
Gender	−0.015	−0.161	0.873	24.744	< 0.001	
Age	0.134	1.445	0.154			
PANSS positive	−0.058	−0.528	0.600			
PANSS negative	−0.118	−1.147	0.256			
PANSS excitement	0.044	0.444	0.659			
CDSS	−0.056	−0.442	0.660			
PSP	0.022	0.185	0.854			
WHOQOL-BREF	0.605	4.974	0.000			

Baseline: Stage 1 adjusted R^2^ = .318, Stage 2 adjusted R^2^ = 0.687, ∆ R^2^ = 0.334, ∆ adjusted R^2^ = 0.369, adjusted R^2^ with all psychological factors = .702

Time point 2: Stage 1 adjusted R^2^ = .283, Stage 2 adjusted R^2^ = 0.491, ∆ R^2^ = 0.194, ∆ adjusted R^2^ = 0.208, adjusted R^2^ with all psychological factors = .539

Note: not all of the psychological factors are reflected in the hierarchical multiple linear regression model above as entering all results in high VIF, refer to text for more details

### Factors associated with the QPR-15

As mentioned previously, the QPR-15 had significant moderate correlations with psychological factors (r_s_ range from 0.521 to 0.687), except for item 6 of the WHOQOL-BREF (r_s_ = 0.472), which was low. The correlations of the QPR-15 with clinical factors were mostly significant but low (r_s_ range from − 0.105 to − 0.544). The only clinical factor that had a moderate correlation with the QPR-15 was depressive symptoms (CDSS: r_s_ = − 0.529 to − 0.544). The PANSS excitement factor at baseline had little correlation with the QPR-15, while the PANSS excitement factor at time point 2 and the PANSS cognitive factor did not have significant correlations with the QPR-15. The rest of the clinical factors had low correlations with the QPR-15.

Next, we entered factors that had significant correlations with the QPR-15 into a hierarchical multiple linear regression model. The regressions were performed separately for the two study visits. Variables were entered into two stages. Clinical factors (PANSS, CDSS and PSP), age and gender were entered first, and psychological factors were entered in the next stage. Clinical factors were entered first as clinical factors and had lower correlation coefficients with the QPR-15 than did psychological factors. The variables were entered simultaneously at each stage. Multicollinearity was detected when all psychological factors were added at stage two of both time points. Thus, only the HHI (VIF 1.276), which had the highest correlation with the QPR-15 at baseline, was entered into the regression model. The same process was performed at time point 2 and yielded the same multicollinearity issues. Thus, only the WHOQOL-BREF (VIF 1.889), which had the highest correlation with the QPR-15 at time point 2, was entered into the regression model.

Regression models are shown in Table 5. The first stage models for the baseline (F change = 5.335, *p* < 0.001; adjusted R^2^ = 0.318) and time point 2 (F change = 4.661, *p* < 0.001; adjusted R^2^ = 0.283) were significant. The strongest predictors among the clinical factors were depressive symptoms (β = − 0.404, *p* = 0.002) and negative symptoms (β = − 0.293, *p* = 0.011) at baseline and depressive symptoms (β = − 0.219, *p* = 0.014) at time point 2. Depressive symptoms (CDSS) continue to be significant at stage two of the baseline when only the HHI was entered into the model. The inclusion of one psychological factor also significantly improved the model variances at baseline (F change = 69.332, *p* < 0.001; adjusted R^2^ = 0.687) and at time point 2 (F change = 24.744, *p* < 0.001; adjusted R^2^ = 0.491) in the second stage.

All the CHIME-related psychological factors were also entered into the model at the second stage of both time points, despite the multicollinearity involved. This procedure was followed to obtain the combined variance accounted for without examining the individual prediction statistics. After controlling for clinical factors, age and gender, the psychological factors representative of the CHIME framework explained an additional 38.4% variance of the QPR-15 at baseline and 22.5% at time point 2. The clinical factors were also no longer statistically significant in predicting the QPR-15.

## Discussion

The current study examined the psychometric properties of the QPR-15 in Singapore, as well as the association between clinical and personal recovery. Our results demonstrate that the QPR-15 has adequate psychometric properties in our socio-cultural setting, possessing CHIME-consistent convergent validity, internal consistency, test-retest reliability and a one-factor structure. To our knowledge, this is the first study that has found empirical evidence for the QPR-15 to represent the CHIME personal recovery framework, as prior QPR studies had excluded the connectedness component and internalized stigma as a subcomponent of identity. Our data also revealed that clinical factors had significant little to moderate (mostly low) correlations with the QPR-15 and explained a significant proportion of variance of the QPR-15; however, this association was no longer statistically significant when all the CHIME-related psychological factors were added into the hierarchical multiple linear regression model. This finding suggested that although psychological factors have a larger contribution to personal recovery than clinical factors, clinical factors still have a complementary role in personal recovery.

The psychometric properties of the QPR-15 obtained from our study are comparable to those found in previous QPR studies. We report an initial factor structure of one factor, which is consistent with the findings of prior QPR studies [25, 27, 28] that had recommended the briefer version of the QPR (15–16 items) but not with the findings of studies providing support for the full version [26, 29, 30]. With regards to convergent validity, the QPR-15 in our study had moderately significant correlation coefficients with subjective scales of psychological factors. This finding is in line with those of other QPR studies (r_s_ = 0.5–0.7). However, it should be noted that among the psychological factors in our study, only the correlation of WHOQOL-BREF item 6 with the QPR-15 (r_s_ = 0.472) was low. Using a single item (item 6) of the WHOQOL-BREF to represent meaning in life might not have been suitable because a more comprehensive measure would have been more valid. The use of a single item could have limited the strength of the correlation between meaning in life and the QPR-15. Nonetheless, Leamy and colleagues [23] reported that 65% of the studies they reviewed had endorsed quality of life as important to the recovery process, categorised under the domain of meaning in life (66% of studies endorsed meaning in life itself). The fact that the QPR-15 showed a moderate correlation with the total WHOQOL-BREF (quality of life) (r_s_ = 0.669) in the current study thus supports its convergent validity with the CHIME domain of meaning in life. Overall, our results provide preliminary evidence for the validity and reliability of the QPR-15 in our socio-cultural setting.

Our results demonstrated that the main clinical predictor of the QPR-15 was depressive symptoms, which is consistent with several studies [3, 7, 8, 11, 60] that examined the relationship between clinical and personal recovery. A recent meta-analysis investigating the association between clinical and personal recovery found a higher mean weighted effect size for affective symptoms compared to symptoms and functioning [60]. Jørgensen et al. [11] also found that the only symptom index that was consistently linked with overall personal recovery over four time points (baseline, 3 months, 6 months and 12 months) was the emotional discomfort factor (depression, anxiety and guilt) of the PANSS. Using the QPR-15, Law et al. [8] found that the QPR-15 scores at time point 2 were predicted by negative emotion (CDSS and negative self-esteem), positive self-esteem, hopelessness and, to a lesser extent, symptoms and functioning. Indeed, our results (Model 1 of both time points in Table 5) suggested that depressive symptoms were the clinical factor with the largest impact on personal recovery. Although depressive symptoms (CDSS) were not significant in the final model (time point 2) of our study, we postulate that the WHOQOL-BREF, through its evaluation of negative feelings, might have captured self-reported depressive symptoms. The results of Roe et al. [3] may also shed some light on how depressive symptoms can affect personal recovery. They found that although the total symptom score did not correlate with subjective recovery (Recovery Assessment Scale; RAS), the dimensions of mood were correlated with the hope domain of the RAS. Therefore, a greater severity of depressive symptoms may reduce one’s level of hope and hence affect one’s personal recovery.

Although our results suggested that psychological factors contributed more to personal recovery than did clinical factors, the relationships between psychological and clinical factors and their effects on personal recovery may be complex. We selected one psychological factor to be entered at stage 2 of the model due to collinearity among the factors. However, we found that inserting different psychological factors into the model produced different effects that clinical factors had on personal recovery. This finding suggests some indirect or interactional relationship amongst the variables, which is congruent with the results of a number of studies [6, 61–64]. For instance, Rossi et al. [61] found that in patients with schizophrenia, avolition (part of negative symptoms) has direct effects on depression, while it also has indirect effects on depression through internalised stigma and resilience. Internalized stigma was related to depression through the mediation of resilience. In addition, Jørgensen et al. [11] found that in addition to depressive symptoms, the relationship of symptom severity with personal recovery did not appear to be generally stable. Negative symptoms were linked to personal recovery at three time points but not one time point, while positive symptoms were linked to subjective recovery at one time point but not three time points. Similarly, we found that negative symptoms were predictive of subjective recovery at one time point (baseline, model 1) but not at time point two of our study. Jørgensen et al. proposed that the result may suggest influence from other factors, such that personal recovery may not be directly impacted by the immediate clinical state. This could also explain why Law et al. found functioning to be of higher significance than depressive symptoms in a cross-sectional study [28] but not in a longitudinal study [8]. Thus, the result of the previously mentioned robust clinical predictor should also be interpreted with caution, as there may be indirect effects from other variables. The role of other clinical factors should not be dismissed. Future longitudinal research should take into account complex indirect or interactional relationships because these relationships can fluctuate due to interrelationships during the time period. This approach will further inform future clinical practice.

Nevertheless, consistent with our hypothesis, our current results showed that compared to psychological factors, clinical factors had lower but significant associations with personal recovery. The size of the correlation for clinical factors was little to moderate (mostly low except for the CDSS, which was moderate; the PANSS excitement correlation was none to little, and the PANSS cognition correlation was none). In contrast, the size of the correlations with psychological factors was moderate (except for WHOQOL-BREF item 6, which had a low correlation). Similarly, the hierarchical multiple linear regression models demonstrated that clinical factors explained a significant proportion of model variance of the QPR-15, albeit a lower proportion than that explained by psychological factors, before psychological factors were entered into the model. This finding is consistent with several studies that have stated that clinical recovery is not synonymous with personal recovery but rather is complementary to it [3, 7, 10, 11, 60]. Therefore, the present results suggest that clinical recovery does play a role in personal recovery.

There were two additional observations made from our results. We found that the HHI (i.e., hope) could explain more of the QPR-15 variance than the clinical factors combined. Perhaps hope is the cornerstone of personal recovery, fuelling one’s motivation to rebuild one’s life and self. It is also noted that self-rated measures of psychological factors can predict self-rated personal recovery compared to objective measures (rated by clinician or trained raters). This finding affirms that there is an aspect of recovery in people with psychosis that is subjective and not assessable by others’ judgement. This is also consistent with the study of Karow et al. [2] that found that service users’ rated remission was only predicted by their self-rated subjective well-being scores but not by objective measures of symptoms and functioning, as rated by psychiatrists.

There are several limitations in this study. The sample size was relatively small; nevertheless, the validation results obtained were consistent with published literature. In addition, a post hoc power analysis revealed that the hierarchical multiple linear regression analysis was adequately powered to detect the observed effects. However, it is advisable to explore the validity of the QPR using its original 22 items in future studies in a larger sample. We recognise that our small sample size was not suitable for factor analysis and hence consider it an initial factor structure using the dimension reduction method (PCA), in which the factor structure may change with larger sample sizes or with factor analysis. Because factor analysis takes into account the underlying structure caused by latent variables, it is a recommended method compared to dimension reduction methods. This approach could answer whether personal recovery is a latent structure that causes the manifest variables (CHIME-related psychological factors) to co-vary. As mentioned above, the usage of a single item (item 6) of the WHOQOL-BREF might not have been valid to represent meaning in life. Hence, future research is recommended to use a more comprehensive measure. Our clinical sample was limited to stable outpatients; therefore, the results might not be generalisable to inpatients or to those who are seriously ill. Our sample was also limited to people with psychosis and might not generalise to other clinical populations. Criterion validity in terms of predictive validity and sensitivity to change could not be examined given the cross-sectional nature of the study. This is an important consideration; as personal recovery measures should be employed as outcome measures to evaluate interventions. Future longitudinal interventional studies would allow for such investigations. Finally, due to the cross-sectional nature of the study, causal inferences cannot be made.

We have identified several future directions. First, there is a need to view recovery within an ecological framework. Although personal recovery is viewed as an individual construct, we need to recognise that individuals exist in a web of relationships with the family, the community and larger socio-political units [65]. Hence, it is necessary to understand the interactions between individual characteristics and environmental factors (such as choice) [23]. Second, as suggested by Leamy et al. [23], we need a deeper understanding of how these individual characteristics of hope, resilience, and empowerment are operating, i.e., how they are ignited and sustained. We have utilised the CHIME framework to guide us in conceptualising personal recovery. However, it remains uncertain if it applies the same way in our population as it did in other socio-cultural settings. Studies have shown or noted that culture and socio-political systems can mediate the operationalisation of the subcomponents of the CHIME framework [66, 67]. Thus, future research is required to evaluate the conceptualisation of personal recovery in Asian socio-cultural settings, the relevance of personal recovery, and the operationalisation of it. Qualitative work has been recommended for these purposes [68]. As mentioned previously, the inter-relationships between clinical and psychological factors and their effect on personal recovery also warrant further work. More research is required before we can effectively capture personal recovery in our socio-cultural setting, hence establishing if the QPR-15 can be used for this purpose and to understand the relationship between personal recovery and clinical recovery. However, it is certain that clinical services will be better informed and thus will improve, both in their delivery and support (psychotherapeutic interventions and rehabilitation), if evaluations of recovery move beyond symptoms and functioning. The evaluation of personal recovery will help provide an index of how service users experience their social environments and themselves as individuals as they make sense of their strengths and challenges [3], since personal recovery is related to personal well-being and social inclusion, which may not be directly captured by clinical recovery [69].

## Conclusions

The QPR-15 possesses adequate psychometric properties in a sample of outpatients with psychosis in Singapore. We have shown that the QPR-15 encompasses the CHIME personal recovery framework, supporting the conclusion made by Leamy and colleagues that the QPR most closely maps to this framework and therefore its usage to evaluate personal recovery. Our study has also clarified the relationship between personal and clinical recovery, supporting our hypothesis that there is a significant but lower association of clinical recovery factors with personal recovery. Therefore, it is essential to consider both forms of recovery as complementary to each other for a more holistic view of recovery in people with psychosis. However, further research is required to better inform clinical implications.

## Additional file

Additional file 1: **Figure S1.** Scree plot for the initial factor structure of QPR-15 in people with psychosis in Singapore (DOCX 212 kb)

## Data Availability

The dataset supporting the conclusions of this article is not available due to ethical restrictions.

## Abbreviations

CDSS: Calgary Depression Scale for Schizophrenia
CHIME: Connectedness, Hope and Optimism about the future, Identity, Meaning in life, and Empowerment
DSM-IV-TR: Diagnostic and Statistical Manual of Mental Disorders, Fourth Edition, Text Revision
HHI: Herth Hope Index
IBM SPSS: International Business Machines Corporation- Statistical Package for the Social Sciences
IMH: Institute of Mental Health
ISMI: Internalized Stigma of Mental Illness
MAP: Minimum Average Partial
PANSS: Positive and Negative Syndrome Scale
PSP: Personal and Social Performance Scale
QPR: Questionnaire about the process of recovery
VIF: Variance Inflation Factor
WHOQOL-BREF: World Health Organization Quality of Life Assessment- Brief form
