# Supplementary figures and images for: An Asian study on clinical and psychological factors associated with personal recovery in people with psychosis
Source: BMC Psychiatry. 2019 Aug 22;19:256. doi: 10.1186/s12888-019-2238-9 (PMC6704510; doi:10.1186/s12888-019-2238-9)

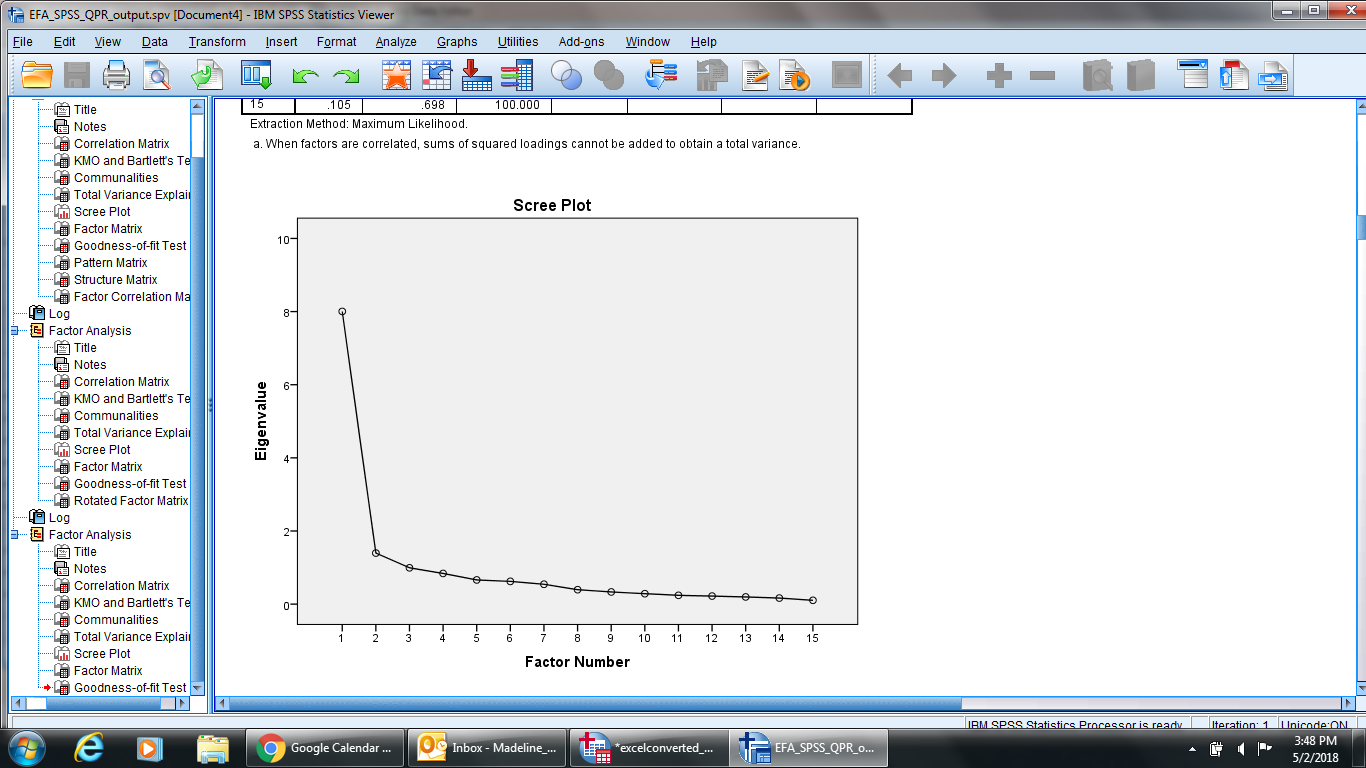

Supplement: Supplementary file 1 — Figure S1. Scree plot for the initial factor structure of QPR-15 in people with psychosis in Singapore (DOCX 212 kb) [file 12888_2019_2238_MOESM1_ESM.docx]
